# A whole-cell tumor vaccine modified to express fibroblast activation protein induces antitumor immunity against both tumor cells and cancer-associated fibroblasts

**DOI:** 10.1038/srep14421

**Published:** 2015-09-23

**Authors:** Meihua Chen, Rong Xiang, Yuan Wen, Guangchao Xu, Chunting Wang, Shuntao Luo, Tao Yin, Xiawei Wei, Bin Shao, Ning Liu, Fuchun Guo, Meng Li, Shuang Zhang, Minmin Li, Kexing Ren, Yongsheng Wang, Yuquan Wei

**Affiliations:** 1 State Key Laboratory of Biotherapy and Cancer Center, West China Hospital, West China Medical School, Sichuan University, Chengdu, China; 2 Department of Immunology, College of Medicine, Key Laboratory of Bioactive Materials, Ministry of Education, Nankai University, Tianjin, China

## Abstract

Cancer-associated fibroblasts (CAFs) are common components of the tumor-suppressive microenvironment, and are a major determinant of the poor outcome of therapeutic vaccination. In this study, we modified tumor cells to express the fibroblast activation protein (FAP), which is highly expressed by CAFs, to potentially improve whole-cell tumor vaccines by targeting both tumor cells and CAFs. Tumor cells were transfected with murine FAP plasmids bearing the cationic lipid DOTAP. Its antitumor effects were investigated in three established tumor models. Vaccination with tumor cells expressing FAP eliminated solid tumors and tumors resulting from hematogenous dissemination. This antitumor immune response was mediated by CD8+ T cells. Additionally, we found that CAFs were significantly reduced within the tumors. Furthermore, this vaccine enhanced the infiltration of CD8+ T lymphocytes, and suppressed the accumulation of immunosuppressive cells in the tumor microenvironment. Our results indicated that the FAP-modified whole-cell tumor vaccine induced strong antitumor immunity against both tumor cells and CAFs and reversed the immunosuppressive effects of tumors by decreasing the recruitment of immunosuppressive cells and enhancing the recruitment of effector T cells. This conclusion may have important implications for the clinical use of genetically modified tumor cells as cancer vaccines.

Stromal cells and their cytokines coordinate critical pathways that play important roles in tumorigenesis, invasion and metastasis^1^. Principal among these cell types is a heterogeneous group of fibroblasts, termed cancer-associated fiassociate (CAFs), which have been shown to play a role in the formation and regulation of the stromal microenvironment^2^. Typically, CAFs promote tumorigenesis and progression via direct cell-to-cell contacts, soluble factors or modification of extracellular matrix components^3^. CAFs are identified based on the expression of the type II membrane dipeptidyl peptidase (DPP) called fibroblast activation protein–α (FAP). These cells exert their immunosuppressive effects by both promoting the recruitment and function of immunosuppressive cells via the secretion of CCL2 and CXCL12 and suppressing effector T cells via the secretion of TGF-β^4^. Moreover, CAFs are genetically more stable than tumor cells, which render CAFs as attractive targets for cancer immunotherapy^5^,^6^.

Whole-cell tumor vaccines have been studied for several decades^7^,^8^,^9^. There are clear advantages to whole-cell vaccination compared with single-target vaccines. First, whole tumor cells provide multiple and unknown tumor-associated antigens (TAAs) that can be targeted by both the innate and adaptive immune systems^10^. Second, whole-cell vaccination may greatly decrease the chance of tumor escape and theoretically dispenses with the need to identify, test and select for immunodominant epitopes^11^. Furthermore, whole tumor cells are more likely to express antigens in a patient-specific manner and to provide patient-matched major histocompatibility complex (MHC) through which TAAs can be recognized. Furthermore, the parallel presentation of both MHC Class I and II antigens facilitates a stronger overall anti-tumor response and long-term CD8+ T cell memory via CD4+ T cells^12^, and this anti-tumor response may be further augmented via the specific modification of the vaccine. Myriad phase I and II clinical trials have demonstrated the safety, tolerability and clinical effects of whole-cell vaccines and the changes in immune function in response to these vaccines. However, as with many other therapeutic vaccination methods, phase III trials of whole-cell vaccination have often failed to demonstrate clinical benefit^13^. Recent studies have suggested that in addition to immune tolerance^14^ and the loss of antigen expression^15^ induced by cancers progression, the immunosuppression within the tumor stromal microenvironment may be a major determinant of the poor efficiency of therapeutic vaccination^16^. There is evidence that the depletion of regulatory T cells (Tregs) may increase the effectiveness of cytokine-secreting tumor-cell vaccines^17^,^18^. Therefore, to improve the clinical benefits of whole-cell tumor vaccines, combining whole-cell vaccination with other anti-immunosuppressive modalities is required.

Based on these findings, we modified a whole-cell tumor vaccine by transducing tumor cells with murine FAP plasmids using the cationic lipid DOTAP to target both tumor cells and CAFs. Then, these tumor cells were irradiated to prevent replication and to enhance antigen presentation. Our results indicated that the whole-cell tumor vaccine modified to express FAP induced strong protective and therapeutic antitumor immunity via CD8+ T-cell-mediated killing. Most importantly, this vaccine suppressed the proliferation and differentiation of M2 macrophages, myeloid derived suppressor cells (MDSCs) and Tregs, which are major components of the immunosuppressive tumor microenvironment. Taken together, our data suggest that immunotherapy targeting both tumor cells and CAFs increases the success of eliminating tumors by dampening the tumor immunosuppressive environment while activating antitumor immunity.

## Results

### pFAP-transfected tumor cells can express active FAP *in vitro*

**Figure 1:**
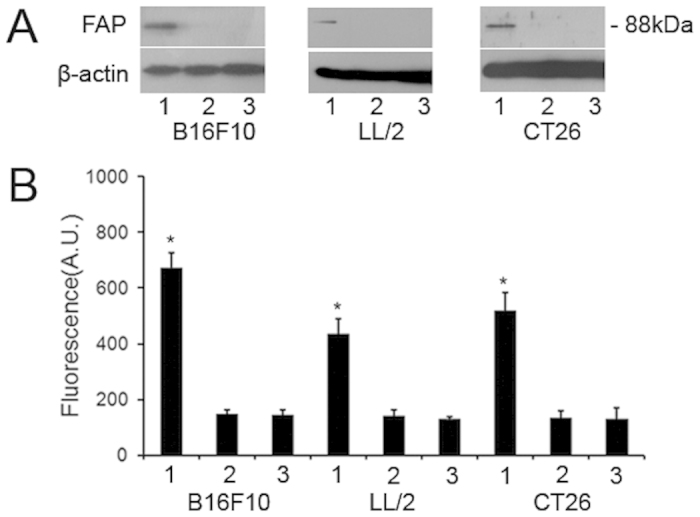
pFAP-transfected tumor cells expressed active FAP *in vitro*. Tumor cells were transfected with the plasmids DNA using the cationic lipid vector DOTAP for 24 hours and were irradiated. (**A**) FAP expression in pFAP-transfected (line 1), pVector-transfected (line 2), and non-transfected (line 3) tumor cells was detected by western blot. β-actin was used as internal control. One cropped representative blot of three is presented and full-length blots are included in the supplementary figure. (**B**) DPP activity in pFAP-transfected (line 1), pVector-transfected (line 2), and non-transfected (line 3) tumor cells (*P < 0.05). Columns, mean (n = 3); bars, SD. Three replicates were done for each treatment.

In the present study, three cell lines were selected: LL/2 (mouse lung cancer), CT26 (mouse colon cancer) and B16F10 (mouse melanoma). These cancers were selected because of their high incidence and because our previous studies showed high vector transfection efficiency in melanoma cells. Tumor cells were transfected with the pFAP expression vector or an empty vector as described in the Materials and methods section. Total protein samples were collected from tumor cells 24 h after transfection and analyzed via western blotting using a pFAP-specific mAb. As shown in Fig. 1A, the 88-kDa murine FAP was detected in the pFAP-transfected tumor cells.

To assess the functional activity of exogenously expressed FAP, we conducted a DPP activity assay before inactivating the cells using radiations. The results revealed significantly higher levels of DPP activity in the pFAP-transfected tumor cells than in the control cells (*P *< 0.05, Fig. 1B). These results indicated that tumor cells transfected with the recombinant pFAP expression vector expressed active FAP *in vitro*.

### Vaccination with tumor cells expressing FAP inhibits transplanted tumor growth in mice

**Figure 2:**
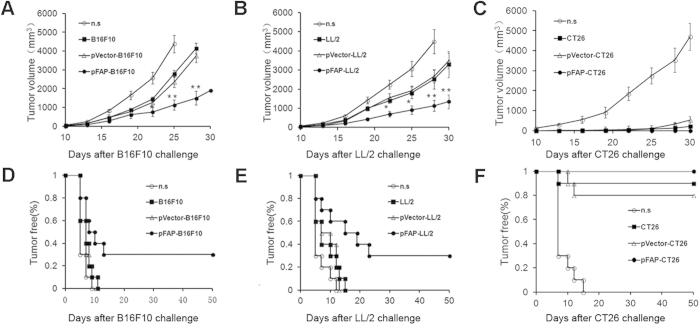
Induction of protective anti-tumor immunity. C57BL/6J and BALB/c mice were immunized with pFAP-transfected, pVector-transfected, or non-transfected tumor cells or n.s. on day −28, −14, −7 and then challenged with 1 × 10^6^ B16F10 cells, LL/2 cells or CT26 cells on day 0. (**a,b**) There was a significant difference in the tumor volume between the mice immunized with pFAP-transfected tumor cells and the control cells in the B16F10 melanoma (B16F10) and Lewis lung carcinoma (LL/2) models. (*P < 0.05 or **P < 0.01; ANOVA). (**d,e**) A significant increase in tumor-free survival of the group immunized with pFAP-transfected tumor cells, compared with other groups, was observed in the B16F10 and LL/2 models (*P *< 0.05). (**c,f**) The immunized mice were nearly tumor-free after challenge with CT26 cells, in contrast to the n.s.-treated mice. Points, mean (n = 10); bars, SD.

To examine whether FAP-expressing tumor cells that were inactivated by irradiation induced systemic anti-tumor immunity, their inhibitory effects on the formation and growth of solid tumors were initially evaluated in a prophylactic setting. Mice were immunized and were challenged with tumor cells as described in the Materials and methods. As shown in Fig. 2, the tumors grew progressively in mice immunized with normal saline (n.s.). Consistent with previous reports, tumor growth was inhibited to a certain extent in all mice immunized with pVector-transfected or nontransfected tumor cells. Interestingly, we detected the suppression of tumor growth in mice immunized with pFAP-transfected tumor cells in B16F10 melanoma (B16F10) and Lewis lung carcinoma (LL/2) models, as both the tumor volume and the tumorigenesis rate were significantly lower in the pFAP-transfected tumor cell-immunized group than in the control groups (Fig. 2A,B,D,E). Furthermore, immunization with irradiated non-modified tumor cells exerted similar suppression of tumor growth in the CT26 colon carcinoma (CT26) model, and all of the immunized mice were tumor-free after tumor cell challenge (Fig. 2C,F).

### Vaccination with tumor cells expressing FAP inhibits tumor growth in tumor-bearing mice

**Figure 3:**
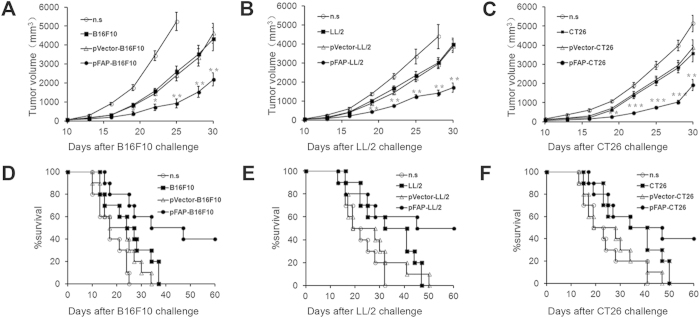
Induction of therapeutic anti-tumor immunity. C57BL/6J and BALB/c mice were treated with pFAP-transfected, pVector-transfected, or non-transfected tumor cells or n.s. on days 5, 12, and 19 after s.c. inoculating 1 × 10^6^ B16F10 cells, LL/2 cells or CT26 cells into mice on day 0. (**a–c**) The tumor volume in the mice immunized with pFAP-transfected tumor cells or a control (*P < 0.05, **P < 0.01 or ***P < 0.001). (**d–f**) A significant increase in the survival of mice immunized with pFAP-transfected tumor cells, compared with the control mice, was observed in the three tumor models (*P *< 0.05). Points, mean (n = 10); bars, SD. Each experimental group was performed twice.

The therapeutic efficacy of the FAP-expressing tumor cell vaccine was evaluated in established tumor models. Mice were treated with the FAP-expressing tumor cell vaccine once weekly for three weeks after the inoculation of tumor cells. As shown in Fig. 3, we found that treatment with pFAP-transfected tumor cell resulted in significant antitumor activity in the B16F10, LL/2 and CT26 models. Tumor growth was significantly retarded and the lifespan was significantly prolonged in mice vaccinated with FAP-expressing tumor cells compared with the control mice. These data indicated that, in spite of the highly significant vaccination activity of non-transduced cells in the CT26 model, irradiated FAP-expressing tumor cells were more efficacious than irradiated cells alone in eliciting anti-tumor immunity in multiple types of tumors.

### Vaccination with tumor cells expressing FAP inhibits lung tumors resulting from hematogenous dissemination in a prophylactic setting

**Figure 4:**
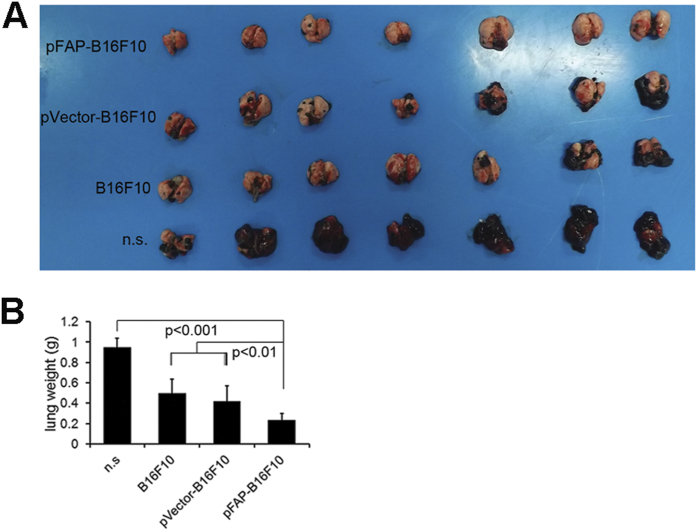
Inhibition of the hematogenous dissemination in lung in a mouse B16F10 melanoma model. C57BL/6J mice were immunized with pFAP-transfected, pVector-transfected, or non-transfected B16F10 cells or n.s., and 5 × 10^5^ B16F10 cells were injected into the tail vein at day 7 after the third immunization. The lungs were weighed and assessed for the presence of hematogenous dissemination when the control mice became moribund. (**a**) Representative images of mouse lungs. (**b**) Average lung weights. The lungs from pFAP-B16F10 cell-immunized mice were significantly smaller than those from mice immunized with pVector-B16F10 or non-transfected B16F10 cells (*P *< 0.01) or treated with n.s. (*P *< 0.001). Points, mean (n = 10).

In addition to promoting the initiation and progression of cancer, CAFs increase the invasiveness of the cancer cells microenvironment via specific communications with cancer cells. Therefore, we examined whether tumors resulting from hematogenous dissemination was also susceptible to immunotherapy using FAP-expressing tumor cells. In the experiment shown in Fig. 4, vaccination with pFAP-transfected tumor cells suppressed the formation and growth of lung tumors resulting from hematogenous dissemination of B16F10 melanoma in a prophylactic setting. The average lung weight was lighter and the number of surface tumors was fewer in the mice immunized with pFAP-transfected tumor cells than in the controls. Furthermore, vaccination with pVector-B16F10 or B16F10 alone also exerted significant inhibitory effects on lung tumors compared with treatment with n.s. (*P *< 0.05). These results indicated that immunotherapy using FAP-expressing tumor cells enhanced the success of elimination of tumor cells after hematogenous dissemination.

### CD8+ T cell-mediated killing may be involved in the anti-tumor immunity induced by whole-tumor cell vaccine expressing FAP

**Figure 5:**
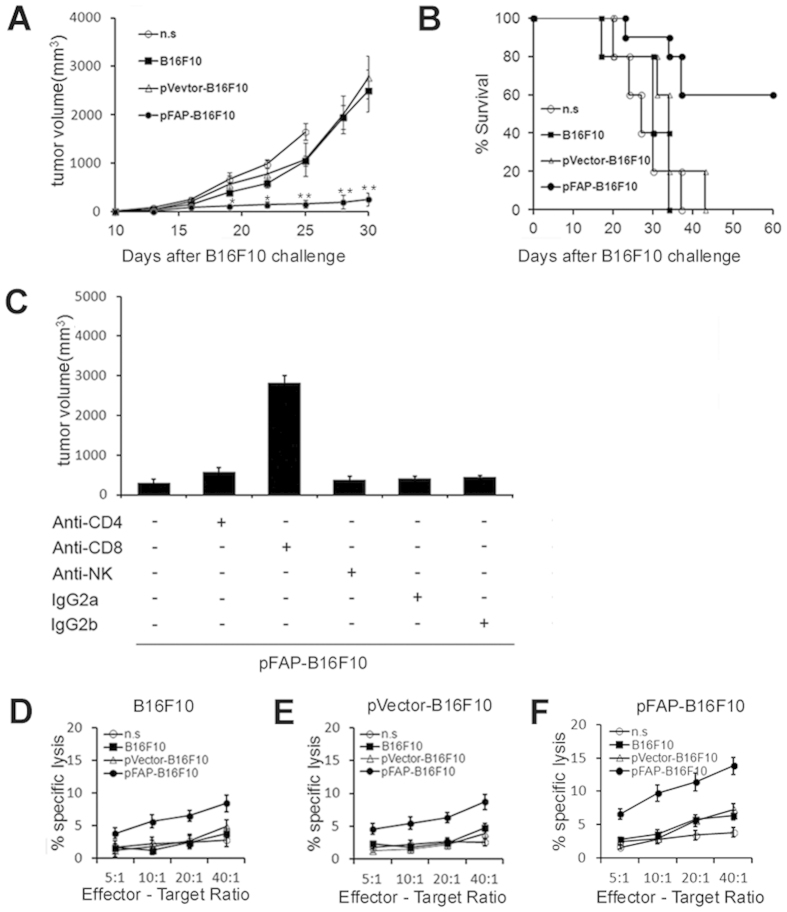
The whole-tumor cell vaccine expressing FAP induces anti-tumor immunity via CD8+ T cell-mediated killing. (**a,b**) The adoptive transfer of T cells *in vivo*. The suppression of s.c. tumor growth (*P < 0.05 or **P < 0.01) and a significant increase in survival (*P *< 0.01) were observed when the recipient mice were injected with T cells from mice immunized with the pFAP-B16F10 vaccine. (**c**) The abrogation of anti-tumor activity via the depletion of immune cell subsets. The depletion of CD8+ T lymphocytes resulted in the complete abrogation of the antitumor activity of the pFAP-B16F10 vaccine. (**d–f**) A representative *in vitro* CTL-mediated cytotoxicity experiment. (**d**) Non-transfected, (**e**) pVector-transfected or (**f**) pFAP–transfected B16F10 cells were used as target cells. T cells isolated from mice immunized with pFAP-B16F10 induced greater cytotoxicity against both FAP-positive and FAP-negative target cells than the control cells (*P *< 0.05) and exerted greater cytotoxicity to FAP-positive cells than to FAP-negative cells.

To explore the potential mechanism by which antitumor activity was elicited by FAP-expressing tumor cells, T cells were isolated from immunized mice and then injected intravenous (i.v.) into C57BL/6J mice. Immunoglobulins were purified from the pooled sera collected from the immunized mice and then injected i.v. into C57BL/6J mice. Tumor growth was markedly suppressed and survival was increased survival in the group that received T cells from pFAP-B16F10 cell-immunized mice (Fig. 5A,B). Furthermore, to explore whether humoral immunity against tumors occurred, an immunoglobulins transfer assay was performed. Adoptive transfer of purified immunoglobulins isolated from pFAP-B16F10 cell-immunized mice resulted in a mild, but not significant, inhibition of tumor growth compared with immunoglobulins isolated from mice immunized with pVector-B16F10 or B16F10 cells (data not shown). This finding indicated that the augmented anti-tumor activity of FAP-expressing tumor cells involved cellular immune responses.

To determine which cell types were critical for this FAP-expressing tumor cell-elicited anti-tumor immune response, mice were depleted of CD4+ or CD8+ T cells or natural killer (NK) cells via the administration of antibodies *in vivo*. Seven days after the final immunization, the mice were challenged with tumor cells injected subcutaneous (s.c.) into the right flank. CD8+ T cells were required for effective vaccination because the depletion of CD8+ T cells before vaccination abrogated the development of systemic immunity, whereas the depletion of CD4+ T cells or NK cells exerted little or no effect (Fig. 5C). These findings suggested that CD8+ T lymphocytes may be involved in the antitumor activity induced by FAP-expressing tumor cells.

To further characterize the requirement of CTL for antitumor immunity, we performed a conventional ^51^Cr-release assay. Splenocytes from mice immunized with pFAP-B16F10, pVector-B16F10 or B16F10 or n.s. were incubated in target cells including untransfected (Fig. 5D), pVector-transfected (Fig. 5E) or pFAP-transfected (Fig. 5F) B16F10 cells, and the percentage of cell lysis was calculated. This rate was the value used to determine target cell auto-lysis. We found that splenocytes from pFAP-B16F10-cell-vaccinated mice did have a higher CTL activity towards the FAP-positive target cells.

### The FAP-modified whole-tumor cell vaccine decreases FAP and collagen expression and increases the infiltration of CD8+ T lymphocytes in tumors

**Figure 6:**
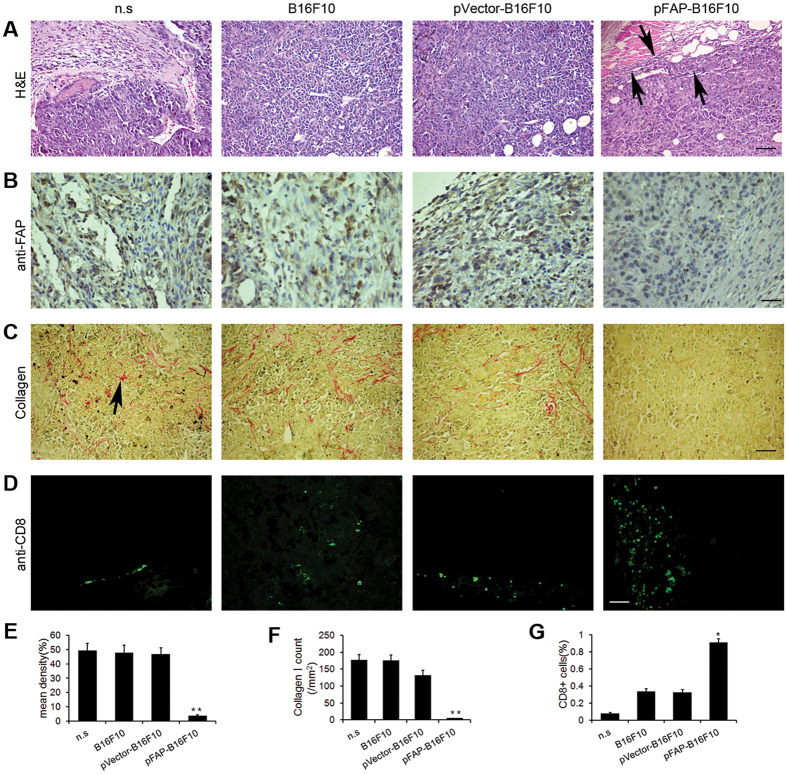
Reduction of FAP-expressing cells and augmentation of lymphocyte infiltration. Sections of the B16F10 tumor tissues were obtained from C57BL/6J mice immunized as described. (**a**) H&E staining of tumor tissues. Increased lymphocyte infiltration (black arrows) and necrotic and apoptotic tumor cells were observed in tumors from pFAP-B16F10 cell-immunized mice. Scale bar, 100 μm. (**b,e**) The expression of FAP in tumor tissues was quantified using Image Pro-Plus according to three parameters: integrated optical density (IOD), area and mean density = IOD sum/area. Significantly decreased expression of FAP was detected in mice vaccinated with pFAP-B16F10 cells (**P < 0.01). Scale bar, 50 μm. (**c,f**) Expression of collagen I in tumor tissues. Significantly decreased collagen expression (black arrows) was detected in the tumors from pFAP-B16F10 cell-immunized mice (**P < 0.01). Scale bar, 100 μm. (**d,g**) Staining of tumor tissues for CD8+ T cells. The number of CD8+ T cells was calculated for each group (*P < 0.05). Scale bar, 50 μm.

As demonstrated using hematoxylin and eosin (H&E) staining, the challenge site of mice vaccinated with pFAP-B16F10 cells contained many eosinophils, monocytes, and lymphocytes, whereas only patches of lymphocytes were detected at the challenge site in mice vaccinated with pVector-B16F10 cells or non-transduced B16F10 cells. Virtually no responding cells were observed in mice vaccinated with n.s. (Fig. 6A).

To determine whether the FAP-modified whole-tumor cell vaccine also targeted CAFs, FAP expression in tumors was initially evaluated using immunohistochemistry. Tumor sections from vaccinated mice were stained with anti-FAP antibodies. As shown in Fig. 6B,E, a significant decrease in the expression of FAP was observed in mice vaccinated with pFAP-B16F10 cells.

Fibroblasts are the primary source of collagen type 1, and the expression of collagen type 1 inversely correlates to the intra-tumoral uptake of various chemotherapeutic drugs^19^. Therefore, we investigated collagen expression by staining tumor sections with Sirius Rose BB. Compared with the mice vaccinated with pVector-transfected or nontransfected B16F10 cells or n.s., the mice vaccinated with pFAP-B16F10 cells displayed significantly decreased expression of collagen type 1 (Fig. 6C). The number of collagen type 1 in six random fields from three different tumors in each group was evaluated, as shown in Fig. 6F. These results are consistent with those of previous studies and suggest that this vaccine exerted its anti-tumor function by directly targeting CAFs and may increase the intra-tumoral uptake of chemotherapeutic agents when used in combination with drug therapy.

To further determine whether infiltrating lymphocytes included CD8+T cells, we stained the tumor sections from vaccinated mice with FITC-conjugated anti-mouse CD8 antibodies. As shown in Fig. 6D, a significant increase in CD8+ T cell infiltration was observed in mice vaccinated with pFAP-B16F10 cells compared with the controls. The number of CD8+ cells was counted, as shown in Fig. 6G. These data confirmed the key role of CD8+ T cells in the anti-tumor response induced by FAP-expressing cells.

### The whole-tumor cell vaccine expressing FAP reduces immunosuppressive cells and enhances CTL recruitment in the tumor microenvironment

**Figure 7:**
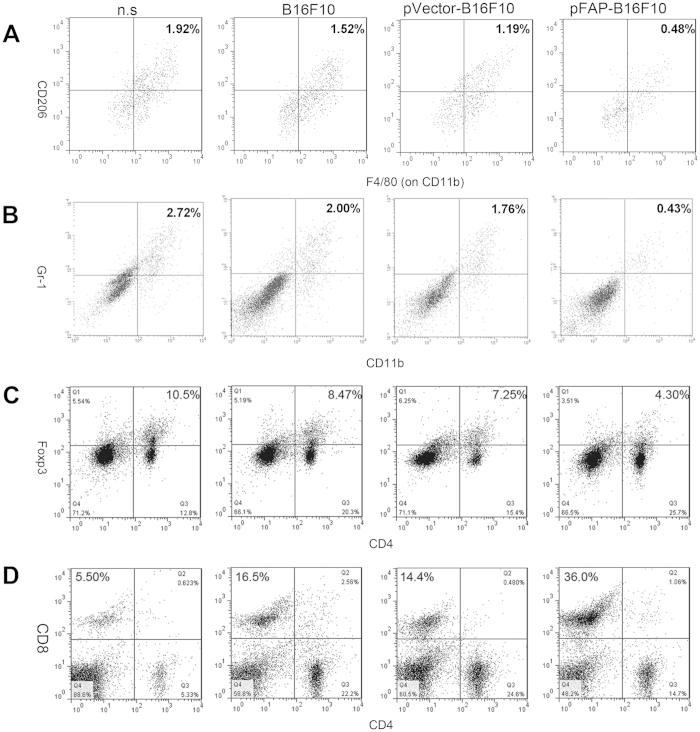
Suppression of immunosuppressive cells and enhancement of CTL recruitment. (**a**) Tumor cell suspensions were stained with antibodies against CD11b (FITC), F4/80 (PE) and CD206 (PerCP) to identify TAMs. (**b**) Tumor cell suspensions were stained with antibodies against CD11b (FITC), and Gr-1 (PE) to identify MDSCs. (**c**) Splenocyte suspensions were stained with antibodies against CD4 (PerCP, cell surface) and FOXP3 (FITC, nuclear) to identify Tregs. (**d**) Splenocyte suspensions were stained with antibodies against CD8 (FITC) and CD4 (PE) to identify T lymphocytes. Vaccination with pFAP-B16F10 cells resulted in significantly decreased numbers of M2 macrophages, MDSCs and Tregs, accompanied by an increased number of CTLs, in the TME (*P < 0.05, **P < 0.01).

Previous studies revealed that stromal cells play a major role in the immunosuppression of tumors and CAFs and, together with tumor-associated macrophages (TAMs), Tregs, MDSCs, and soluble factors produced by suppressor cells, contribute to cancer-induced immunosuppression^20^. To determine whether the FAP-expressing tumor cell vaccine, which targets both CAFs and tumor cells, reverses the suppressive effects of tumors on the immune system by changing the immune cell milieu of the tumor microenvironment (TME), we performed flow cytometry to identify TAMs, MDSCs, Tregs and effector T cells (e.g., CD8+ CTLs) in the primary tumors. As shown in Fig. 7, vaccination with pFAP-B16F10 cells markedly reduced the relative percentage of M2 macrophages, MDSCs, and Tregs. By contrast, the relative percentage of CD8+ CTLs in the TME was significantly increased. These results are consistent with those of previous studies and indicate that CAFs act as key modulators of the immune cell milieu in the TME and that vaccination with FAP-expressing cells effectively suppresses the recruitment of pro-tumor immune cells while enhancing the recruitment of anti-tumor immune cells.

### Observation of possible side effects

The vaccinated animals were monitored for possible side effects for >10 months. No adverse effects were found based on gross measures of health such as weight loss, ruffling of fur, behavior, life span or feeding. In addition, no pathologic changes in the lung, liver, kidney, spleen or heart were detected based on microscopic examination (Supplementary Figure S1).

## Discussion

In the current study, we have shown that the genetic modification of a whole-tumor cell vaccine to express FAP resulted in slowing tumor growth and increased survival in mice inoculated with multiple tumor types. Previous studies have suggested that whole-cell tumor vaccines are more effective clinically when used in combination with other modalities, such as treatment with soluble cytokines, immunomodulatory drugs, or anti-angiogenics, chemotherapy and radiotherapy^7^. In this study, we expanded the scope of the whole-tumor cell vaccine to antigens expressed in CAFs, the predominant component of the stroma. In addition, we showed that this approach could be of some use in the prophylactic setting.

FAP is an essential protease expressed in CAFs in more than 90% of human epithelial carcinomas including those of the breast, lung, colorectum, and ovary^21^. The transient overexpression of FAP is also be detected during wound healing and in some fetal mesenchymal tissues^22^. FAP displays both DPP and endopeptidase activity, including collagenolytic activity that degrades gelatin and type I collagen^23^. High stromal levels of FAP have been associated with aggressive progression and metastasis or recurrence of colon cancer^24^. The abrogation of the enzymatic activity of FAP attenuates tumor growth^24^. The use of FAP inhibitory antibodies attenuated tumor growth in an animal model, and treatment with a monoclonal antibody (mAb) against FAP has been shown to inhibit tumor growth in clinical trials^25^. Therefore, the modified whole-tumor cell vaccine expressing FAP displays several attractive characteristics, including:

1. As tumor cells are genetically unstable, tumor antigens are not ideal targets for anticancer therapy. By contrast, CAFs are genetically more stable, exhibit limited proliferative capacity and reduce the rate of immune evasion^5^,^6^.
2. Antigen presentation by stromal fibroblasts to the T cell receptor complex is not impaired by the downregulation of MHC class I antigen expression^19^,^26^,^27^.
3. As discussed regarding immunotherapy, targeting both cancer cells and FAP-expressing cells to deliver two distinct and potentially synergistic treatment modalities may provide a new approach for the treatment of cancer.
4. The expression of many stromal components such as FAP is expressed on many tumors. As such, these components can be targeted in most, if not all, cancer patients^28^,^29^.
5. Fibroblasts are the primary source of collagen type 1, and the expression of collagen type 1 inversely correlates to the intra-tumoral uptake of various chemotherapeutic drugs. Immunotherapy against CAFs in combination with drug therapy increases the intra-tumoral uptake of chemotherapeutic agents^23^,^30^,^31^,^32^.

However, hosts normally do not develop antibodies against FAP. This may suggest that the inactivation process using radiations modify FAP in a way that induces the production of antibodies, and these antibodies cross-react with the intact FAP found on cancer cells of the host^33^. However, further study is necessary to explore this issue.

Immunotherapies consisting of cell vaccines engineered using non-viral vectors expressing tumor antigens represent promising strategies for safe antitumor therapies, as well as possibilities in the prophylactic setting^34^. This study extends the use of this approach to stromal tumor targets. Tumor cells were transfected with a recombinant murine FAP plasmid using the cationic lipid, DOTAP, and were irradiated to prevent their replication and to enhance antigen presentation. We found that this pFAP transfectant expressed bioactive FAP *in vitro*. In this assay, we used murine melanoma B16F10 cells, Lewis lung carcinoma LL/2 cells and colon carcinoma CT26 cells to generate tumor models to assess the anti-tumor effects of the FAP-modified tumor cells. In spite of the highly significant suppressive effects of irradiated non-transfected tumor cells from tumor growth in the CT26 colon carcinoma model, the pFAP-transfected tumor cells exhibited enhanced inhibitory effects on tumor onset and growth in multiple tumor models, implying that the FAP-expressing tumor cells could elicit strong protective and therapeutic anti-tumor immunity and enhance the efficacy of immunotherapy based on whole-tumor cell vaccines. One possible explanation for this result is that CT26 cells are inherently immunogenic when inactivated by irradiation and that the level of anti-tumor immunity achieved using irradiated non-transduced cells was similar to those using FAP-transduced cells. Moreover, evidence from both preclinical and clinical studies suggests that treatment with tumor-cell vaccines may be more effective at earlier stages, which may contain a less immunosuppressive environment than at later stages, and which may facilitate the efficacy of these vaccines^7^. Therefore, cancer cells with high immunogenic potential could be used as vaccine in the prophylactic setting^35^,^36^ since some proteins like FAP are mostly expressed in cancers^26^,^27^.

CAFs promote tumor growth and metastasis via their role as key modulators of immune polarization in the TME^37^. Because FAP is expressed in CAFs, but not in the tumor cells used in this study, we hypothesize that the observed tumor inhibition may have occurred, in part, due to CAF-mediated modulation of the immune TME and direct antitumor immunity. Although no *in vitro* experiment examining the targets of CAFs in this vaccine was performed, by evaluating the expression of FAP and collagen type 1 in the tumors, we demonstrated that the decrease of FAP and collagen type 1 occurred in pFAP-B16F10 cell-vaccinated mice, implying the specificity of this vaccine for CAFs. As the process of tumor invasion and metastasis is dependent on tumor-stromal interactions, the elimination of cancer cell variants by the immune system is presumably due to “bystander killing” that is secondary to the elimination of the stroma. Recent studies have suggested that immunization of tumors against fibroblasts in tumors unmasks the immune response to cancer^33^,^38^,^39^ and that the depletion of FAP-expressing cells caused rapid hypoxic necrosis of both cancer and stromal cells in immunogenic tumors^16^. By immunologically targeting both tumor cells and FAP-expressing cells for destruction, this study has confirmed and extended these findings.

In addition, these results showed that the selective depletion of effector cells *in vivo* indicated that CD8+ T cells, but not CD4+ T cells or NK cells, was required for the anti-tumor response. Non-specific immune responses were unlikely, because the cytotoxic effects mediated by CD8+ T cells *in vitro* were significantly enhanced against target cells overexpressing the FAP antigen, which is consistent with other studies^23^,^40^. Taken together, these findings suggest that immunotherapy using an FAP-expressing vaccine induces immunity to both TAAs and FAP and stimulates the formation tumor-specific CTLs, predominantly CD8+ cells, which are directed toward antigens associated with the patient’s cancer.

Previous studies revealed that the TME favors immune-suppressive regulators rather than immune effectors and that tumors escape from immune attack via a variety of complementary immunosuppressive mechanisms^4^,^41^. In addition to the increased expression of immunosuppressive molecules (e.g., FasL) and cytokines (e.g., IL-10 and TGF-β), tumor cells can directly escape T-cell recognition by downregulating MHC class I, by disabling other components of the antigen-processing machinery, and by upregulating surface ligands including PD-L1 and other ligands to inhibitory T-cell receptors, which mediate T-cell anergy (or exhaustion). Moreover, a variety of leukocyte subsets that infiltrate the tumors are also able to block anti-tumor CTL- or NK/T cell-mediated killing of aberrant cells. Among these cell types, TAMs may drive multiple pro-tumor processes, including immunosuppression, angiogenesis, and the direct secretion of tumor growth factors^42^. In addition, Tregs may exert their immunosuppressive effects by both directly interacting with tumor cells via the expression of surface molecules such as CTLA-4, and via the secretion of cytokines such as IL-10 and TGF-β^43^. Additionally, MDSCs suppress T and NK cell activation, likely via several mechanisms including nitric oxide, reactive oxygen species, arginase, IL-10 and TGF-β. There have also been reports that MDSCs may specifically induce the expansion of Tregs, reduce the number of effector T cells (e.g., CD8+ CTLs) and promote the Th2 response^44^. Moreover, CAFs perform important immunosuppressive functions by promoting the recruitment and function of immunosuppressive cells via the secretion of CCL2 and CXCL12, and by suppressing effector T cells via the secretion of TGF-β.

Currently, much research about cancer immunotherapy has focused on immunosuppressive markers, targets, and combinational approaches, demonstrating that dampening the tumor immunosuppressive environment while activating innate antitumor immunity may represent an effective approach to cancer treatment^45^. Previous studies have shown that a DNA vaccine targeting FAP induced a shift in local immunity from Th2 to Th1, which favors the destruction of malignant cells, in a murine breast cancer model^39^. During our investigation of the immune-related changes in the TME, we found significantly suppressed recruitment of MDSCs, M2 macrophages and Tregs, and enhanced recruitment of CD8+ T cells in pFAP-B16F10 cell-vaccinated mice. Thus, it appears that the FAP-expressing tumor cell vaccine may achieve maximal anti-tumor effects, as it targets both CAFs and tumor cells for elimination. However, further studies are required to determine the molecular mechanisms by which FAP-modified tumor cell vaccines are involved in the crosstalk between CAFs, tumor cells and infiltrating immune cells.

Taken together, to our knowledge, this is the first report showing that a FAP-modified whole-tumor cell vaccine induced strong protective and therapeutic anti-tumor immunity via CD8+ T cell-mediated killing. Notably, the FAP-expressing tumor cells directly targeted both tumor cells and CAFs in tumors and reversed the immunosuppressive effects of tumors by reducing the recruitment of immunosuppressive cells and enhancing the recruitment of effector T cells. This conclusion has important implications for the clinical use of genetically modified tumor cells as cancer vaccines, but further study is necessary before the use of the present technique in clinical trials.

## Materials and Methods

### Animals and cell lines

C57BL/6J and BALB/C mice were purchased from the West China Experimental Animal Center. All of the animal experiments were approved and conducted in accordance with the Animal Care and Use Committee of Sichuan University. Murine melanoma B16F10 cells and Lewis lung carcinoma LL/2 cells were cultured in DMEM, and colon carcinoma CT26 cells were cultured in RPMI 1640. Both media were supplemented with 10% (v/v) fetal bovine serum.

### Tumor cell vaccine preparation

Plasmids containing murine FAP genes were constructed as previously described^33^. The reconstructed plasmid or empty plasmid vector was transfected into the B16F10, LL/2 or CT26 cells using the cationic lipid DOTAP for transient transfection^34^, and we used a mass ratio of 4:1 (DOTAP:DNA). For *in vivo* use, tumor cells were irradiated (100 Gy) for 24 hours after transfection and washed three times in PBS to remove the medium. Other samples that were not transfected were treated similarly.

### Western blot

The expression of plasmid DNA was detected in transfected tumor cells using western blotting before inactivating the cells using radiations. pFAP-transfected, pVector-transfected and untransfected tumor cells were lysed in RIPA buffer on ice. Then, cell lysates were centrifuged at 13,000 g at 4 °C for 20 min. The protein concentration in the cell lysate was measured using the Lowry method. Equal amounts of protein were resolved by SDS-PAGE and transferred onto polyvinylidene difluoride (PVDF) membranes (Amersham Bioscience, Piscataway, NJ). After incubation with a polyclonal rabbit anti-murine FAP antibody (mAb; Abcam, Cambridge, UK) and secondary antibody, the protein bands were visualized using an enhanced chemiluminescent substrate to horseradish peroxidase (Amersham, Piscataway, NJ). β-actin was used as internal control.

### DPP activity assay

The DPP activity of these protein products produced by tumor cells was performed as previously described^33^. Briefly, an immunocapture assay was performed using Ala-Pro-7-amido-4- trifluoromethylcoumarin (Ala-Pro-AFC) as a substrate (Bachem, Torrance, CA, USA). Fluoronunc MaxiSorb 96-well plates (Nunc, Roskilde, Denmark) were coated overnight at 4 °C with 100 μg/mL anti-FAP antibodies and washed with phosphate-buffered saline containing 0.1% Tween20 (PBST), blocked in 5% bovine serum albumin for 1 h at room temperature. Total protein from pFAP-transfected, pVector-transfected and untransfected tumor cells was extracted using detergent protein extraction reagent (Pierce Biotechnology, Rockford, IL, USA), according to the manufacturer’s instructions. The protein concentration was measured using the Lowry method. Approximately 1 mg of total protein extracts was added to each well and incubated for 1 h, followed by 10 washes with PBST. The DPP activity was assessed by cleavage of 0.25 mmol/L Ala-Pro-AFC for 1 h at room temperature. Release of the free AFC fluorescent substrate was detected using a cytofluor fluorimeter (Labsystems, Helsinki, Finland) with 396 nm excitation and 490 nm emission wave lengths.

### Immunization and tumor models

The mice were immunized with 1 × 10^6^ irradiated pFAP-transfected, pVector-transfected, or non-transfected tumor cells or n.s. via subcutaneous (s.c.) injection into the bilateral inguinals and the unilateral axillary on day −28, −14 and −7. Seven days after the final immunization, the mice were inoculated with 1 × 10^6^ tumor cells s.c. into the right flank. To investigate the therapeutic effect of these vaccines on established tumors, the mice were treated via s.c. injection of 1 × 10^6^ irradiated pFAP-transfected, pVector-transfected, or non-transfected tumor cells or n.s. on days 5, 12, and 19 after the s.c. inoculation of 1 × 10^6^ live tumor cells on day 0. Tumor growth was monitored, and the tumor volume was calculated according to the following formula: width^2 ^× length × 0.52. Due to the death of some mice, the tumor volume on day 28 in the control groups only includes the data from the surviving mice.

To determine the efficacy of the tumor cell vaccines in the models with tumors resulting from hematogenous dissemination, 5 × 10^5^ B16F10 cells were injected into the tail vein of each C57BL/6J mouse on day 7 after the third immunization^46^. Twenty days later, the mice were killed when the control mice became moribund and the lungs were weighed.

### Adoptive transfer *in vivo*

C57BL/6J mice were immunized, and T cells were isolated according to previously published methods^47^. Freshly isolated T lymphocytes (1 × 10^7^) were injected into the tail vein of the recipient mice on the second day after B16F10 cell challenge. Immunoglobulins were purified from the pooled sera collected from the immunized or control mice as previously described^48^. Purified immunoglobulins were transferred intravenous (i.v.) at 50 mg/kg per mouse 1 day before the mice were challenged with B16F10 cells, after which they were administered twice per week for 3 weeks. Tumor growth and survival were monitored in both of the experiments.

### *In vivo* depletion of immune cell subsets

Immune cell subsets were depleted as described^49^. Briefly, the anti-mouse CD4 (clone GK 1.5, rat IgG), anti-mouse CD8 (clone 2.43, rat IgG), or anti-natural killer (clone PK136) mAb or an isotype control was administered intraperitoneally (i.p.) at a dose of 500 μg/mouse 1 day before immunization and, subsequently, twice per week for 3 weeks. Then, the mice were challenged with B16F10 tumor cells after the third immunization. The tumor size was measured 25 days after challenge.

### *In vitro* cytotoxicity assay

To determine whether CTL mediate FAP-specific cytotoxicity, a ^51^Cr release assay was performed as described previously^49^,^50^. Splenocytes were isolated from mice immunized with pFAP-transfected, pVector-transfected, or non-transfected B16F10 cells or n.s. T lymphocytes isolated from single-cell suspensions using Nylon Fiber Column T (L-Type; Wako, Tokyo, Japan) were used as CTL effector cells, whereas the pFAP-transfected, pVector-transfected and non-transfected B16F10 cells were used as specific target cells. The effector cells and ^51^Cr-labeled target cells were seeded on 96-well microtiter plates at different effector:target ratios and were incubated for 4 hours. The CTL activity was calculated using the following formula: % lysis = [(experimental release − spontaneous release)/(maximal release − spontaneous release)] × 100.

### Immunohistochemistry and cell staining

Tumor tissues were histologically analyzed using H&E staining. To assess FAP expression in the tumor stroma, the tumor sections were first stained with an anti-FAP primary antibody (Bender MedSystems, Vienna, Austria) and then stained with streptavidin-labeled biotin reagents (DAKO LSAB kit, peroxidase; DAKO, Carpinteria, CA, USA). To evaluate collagen expression, Sirius Rose BB (Sigma; St Louis, MO, USA) staining was performed according to the manufacturer’s protocol. To stain CD8+ T cells, tumor sections were stained with an anti-mouse CD8 antibody (FITC-labeled; eBioscience, San Diego, CA, USA), according to the manufacturer’s instructions. All images were analyzed using Image Pro Plus software (Media Cybernetics, Bethesda MD, USA) as previously described^51^.

### Flow cytometry

Flow cytometry was used to determine whether FAP-modified tumor cell vaccination correlated to changes in the immune cell milieu of the TME. Mice were immunized and challenged with B16F10 cells, as described. Primary tumor cell suspensions were isolated from the tumor-bearing mice, and splenocytes were isolated from the lymph nodes near the tumor. Anti-CD11b (FITC-labeled; BD Biosciences), anti-F4/80 mAbs (PE-labeled; BD Biosciences) and anti-CD206 (PerCP-labeled; BD Biosciences) were used to detect TAMs. Anti-CD11b (FITC-labeled; BD Biosciences, Stockholm, Sweden) and anti-Gr1 mAbs (PE-labeled; BD Biosciences) were used to detect MDSCs. For Foxp3 intracellular staining, we used an APC-conjugated anti-mouse/rat Foxp3 staining kit (eBioscience) according to the manufacturer’s instructions. Anti-CD8 (FITC-labeled; BD Biosciences) and anti-CD4 mAbs (PE-labeled; BD Biosciences) were used to detect effector T cells. The fluorescently stained cells were detected using a BD FACSCalibur flow cytometer (BD Biosciences), and the data were analyzed using FlowJo software (Tree Star, Inc, Ashland, OR, USA). The results are representative of 3 separate experiments.

### Evaluation of possible adverse effects

To investigate the potential toxicity of each vaccine to mice during the treatment, the mice were continuously observed for gross measures of health such as weight loss, ruffling of fur, life span, behavior, and feeding. The heart, liver, spleen, lung and kidney tissues were also analyzed using H&E staining.

### Statistical analysis

SPSS 17.0 was used for statistical analysis. The significance of the results for all of the experiments was determined via ANOVA and the log-rank test. The findings were regarded as significant if P < 0.05.

## Additional Information

**How to cite this article**: Chen, M. *et al.* A whole-cell tumor vaccine modified to express fibroblast activation protein induces antitumor immunity against both tumor cells and cancer-associated fibroblasts. *Sci. Rep.* **5**, 14421; doi: 10.1038/srep14421 (2015).

## Supplementary Material

Supplementary Information
