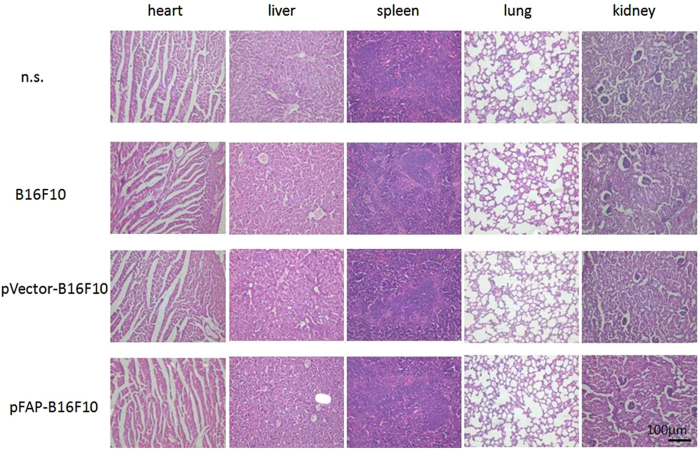# Corrigendum: A whole-cell tumor vaccine modified to express fibroblast activation protein induces antitumor immunity against both tumor cells and cancer-associated fibroblasts

**DOI:** 10.1038/srep46841

**Published:** 2017-10-20

**Authors:** Meihua Chen, Rong Xiang, Yuan Wen, Guangchao Xu, Chunting Wang, Shuntao Luo, Tao Yin, Xiawei Wei, Bin Shao, Ning Liu, Fuchun Guo, Meng Li, Shuang Zhang, Minmin Li, Kexing Ren, Yongsheng Wang, Yuquan Wei

Scientific Reports 5: Article number: 14421; 10.1038/srep14421 published online: 09 23 2015; updated: 10 20 2017.

In the Supplementary Information of our Article panels are duplicated in Figure S2. The panel for B16F10 in spleen is duplicated as the panel for pVector-B16F10 in spleen. The panel for n.s. in kidney is duplicated as the panel for B16F10 in kidney. These duplications are a result of mistakes made during the preparation of Figure S2. The correct Figure S2 appears below as Figure 1.

## Figures and Tables

**Figure 1:**