# Author Correction: A whole-cell tumor vaccine modified to express fibroblast activation protein induces antitumor immunity against both tumor cells and cancer-associated fibroblasts

**DOI:** 10.1038/s41598-020-72743-8

**Published:** 2020-09-21

**Authors:** Meihua Chen, Rong Xiang, Yuan Wen, Guangchao Xu, Chunting Wang, Shuntao Luo, Tao Yin, Xiawei Wei, Bin Shao, Ning Liu, Fuchun Guo, Meng Li, Shuang Zhang, Minmin Li, Kexing Ren, Yongsheng Wang, Yuquan Wei

**Affiliations:** 1 State Key Laboratory of Biotherapy and Cancer Center, West China Hospital, West China Medical School, Sichuan University, Chengdu, China; 2 Department of Immunology, College of Medicine, Key Laboratory of Bioactive Materials, Ministry of Education, Nankai University, Tianjin, China

Correction to: *Scientific Reports* 10.1038/srep14421, published online 23 September 2015

This Article contains errors.

Figure S2 contains duplicated panels. In the original image, the panel for B16F10 in spleen is duplicated as the panel for pVector-B16F10 in spleen. This duplication is a result of errors in figure assembly.

The previous correction of this figure, published on 20 October 2017, also contains duplications introduced during the assembly of the revised figure: panel n.s. for spleen is duplicated as panel pFAB-b16F10 for spleen.

The corrected Figure S2, addressing both errors, appears below as Figure 1.

**Figure 1:**
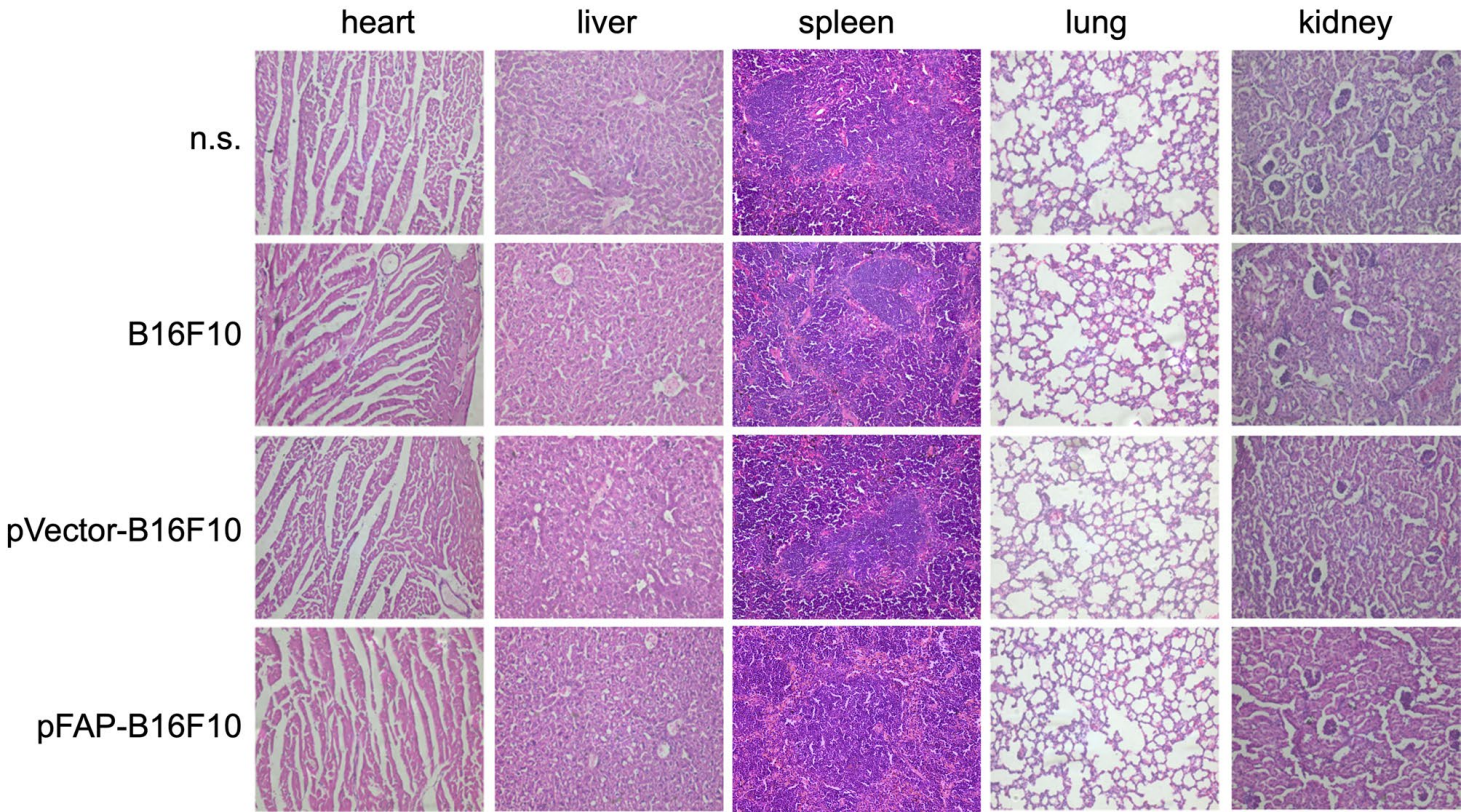
The pFAP-transfected tumor cell vaccine did not cause obvious pathologic changes in normal tissues. Paraformaldehyde fixed organs (heart, liver, spleen, lungs and kidneys) were processed for paraffin embedding and then stained by hematoxylin and eosin. Images shown are representatives from each group. Scale bars represent 100 μm.

