# Supplementary material for: A whole-cell tumor vaccine modified to express fibroblast activation protein induces antitumor immunity against both tumor cells and cancer-associated fibroblasts
Source: Sci Rep. 2015 Sep 23;5:14421. doi: 10.1038/srep14421 (PMC4585784; doi:10.1038/srep14421)
Supplement: Supplementary Information [file srep14421-s1.doc]

**Supplementary information**

A whole-cell tumor vaccine modified to express fibroblast activation protein induces antitumor immunity against both tumor cells and cancer-associated fibroblasts

Authors: Meihua Chen^1^, Rong Xiang^2^, Yuan Wen^1^, Guangchao Xu^1^, Chunting Wang^1^, Shuntao Luo^1^, Tao Yin^1^, Xiawei Wei^1^, Bin Shao^1^, Ning Liu^1^, Fuchun Guo^1^ , Meng Li^1^, Shuang Zhang^1^, Minmin Li^1^, Kexing Ren^1^, Yongsheng Wang^1^*, Yuquan Wei^1^*

Authors’ affiliations: ^1^State Key Laboratory of Biotherapy and Cancer Center, West China Hospital, West China Medical School, Sichuan University, Chengdu, China.

^2^ Department of Immunology, College of Medicine, Key Laboratory of Bioactive Materials, Ministry of Education, Nankai University, Tianjin, China.

*Corresponding author: Yuquan Wei or Yongsheng Wang, State Key Laboratory of Biotherapy, West China Hospital, West China Medical School, Sichuan University, Gaopeng Street, Keyuan Road 4, Chengdu, 610041, China. Phone: 86-028-85164063; Fax: 86-028-85164060.E-mail: yqwei@vip.sina.com or [wangys75@gmail.com](mailto:wangys75@gmail.com)


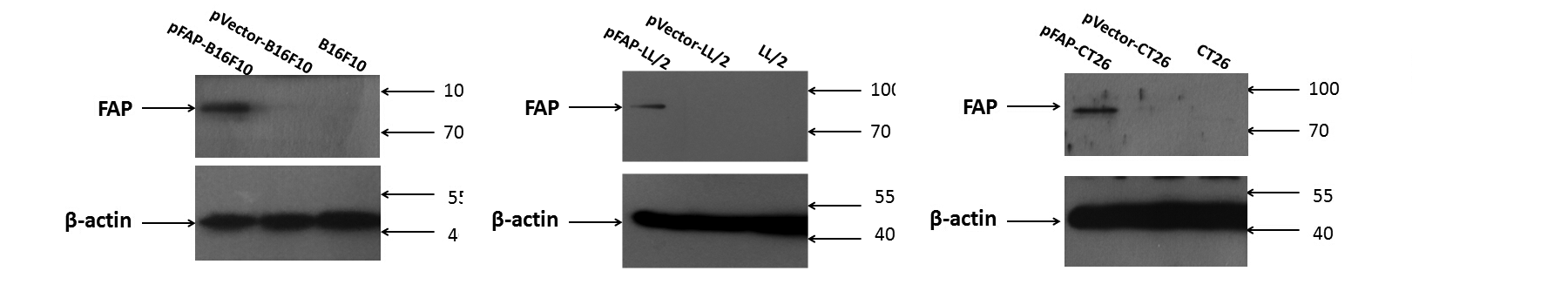


Supplementary FigureS1. The exression of FAP was tested by western blotting in pFAP-transfected, pVector-transfected, and non-transfected tumor cells. Tumor cells were transfected with the plasmids DNA using the cationic lipid vector DOTAP for 24 hours and were irradiated. FAP expression was detected by western blot. β-actin was used as internal control . These cropped blots are used in the main figure (Figure 1) and these full-length blots are included in the supplementary figure.


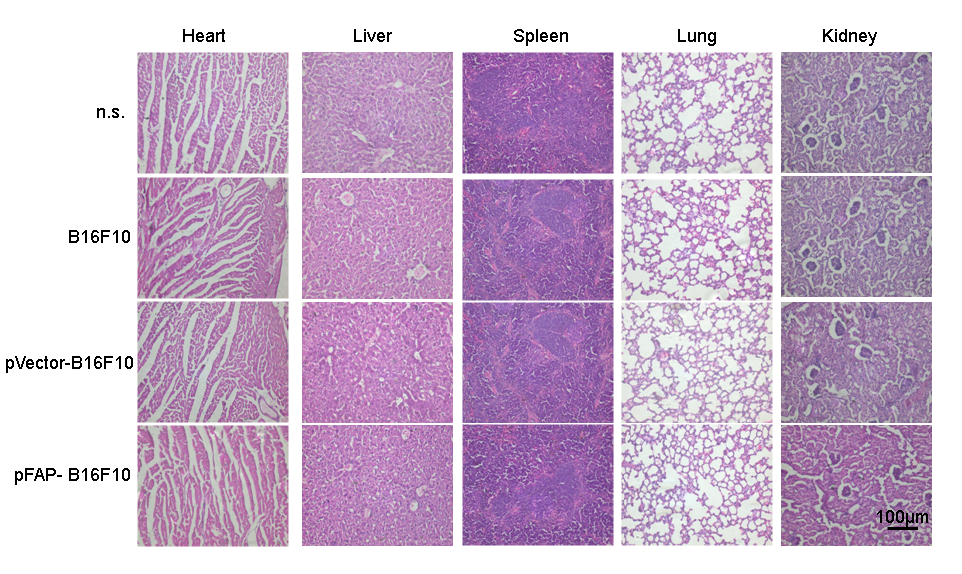


Supplementary FigureS2. The pFAP-transfected tumor cell vaccine did not cause obvious pathologic changes in normal tissues. Paraformaldehyde ﬁxed organs (heart, liver, spleen, lungs and kidneys) were processed for paraﬃn embedding and then stained by hematoxylin and eosin. Images shown are representatives from each group. Scale bars represent 100 μm.
